# Resveratrol in the Hypothalamic Paraventricular Nucleus Attenuates Hypertension by Regulation of ROS and Neurotransmitters

**DOI:** 10.3390/nu14194177

**Published:** 2022-10-07

**Authors:** Jie Qi, Li-Yan Fu, Kai-Li Liu, Rui-Juan Li, Jin-An Qiao, Xiao-Jing Yu, Jia-Yue Yu, Ying Li, Zhi-Peng Feng, Qiu-Yue Yi, Hong Jia, Hong-Li Gao, Hong Tan, Yu-Ming Kang

**Affiliations:** 1 Department of Physiology and Pathophysiology, School of Basic Medical Sciences, Xi’an Jiaotong University Health Science Center, Xi’an 710061, China; 2 Institute of Cardiovascular Sciences, Translational Medicine Institute, Xi’an Jiaotong University Health Science Center, Xi’an 710061, China; 3 Key Laboratory of Environment and Genes Related to Diseases, Xi’an Jiaotong University, Ministry of Education, Xi’an 710061, China; 4 Department of Infectious Diseases, The Second Affiliated Hospital, Air Force Military Medical University, Xi’an 710038, China; 5 Institute of Pediatric Diseases, Xi’an Children’s Hospital, Xi’an 710002, China; 6 College of Life Sciences, Northwest University, Xi’an 710069, China

**Keywords:** resveratrol, high blood pressure, PVN, SIRT1, ROS, excitatory and inhibitory neurotransmitters

## Abstract

Background: The hypothalamic paraventricular nucleus (PVN) is an important nucleus in the brain that plays a key role in regulating sympathetic nerve activity (SNA) and blood pressure. Silent mating-type information regulation 2 homolog-1 (sirtuin1, SIRT1) not only protects cardiovascular function but also reduces inflammation and oxidative stress in the periphery. However, its role in the central regulation of hypertension remains unknown. It is hypothesized that SIRT1 activation by resveratrol may reduce SNA and lower blood pressure through the regulation of intracellular reactive oxygen species (ROS) and neurotransmitters in the PVN. Methods: The two-kidney one-clip (2K1C) method was used to induce renovascular hypertension in male Sprague-Dawley rats. Then, bilaterally injections of vehicle (artificial cerebrospinal fluid, aCSF, 0.4 μL) or resveratrol (a SIRT1 agonist, 160 μmol/L, 0.4 μL) into rat PVN were performed for four weeks. Results: PVN SIRT1 expression was lower in the hypertension group than the sham surgery (SHAM) group. Activated SIRT1 within the PVN lowered systolic blood pressure and plasma norepinephrine (NE) levels. It was found that PVN of 2K1C animals injected with resveratrol exhibited increased expression of SIRT1, copper-zinc superoxide dismutase (SOD1), and glutamic acid decarboxylase (GAD67), as well as decreased activity of nuclear factor-kappa B (NF-κB) p65 and NAD(P)H oxidase (NOX), particularly NOX4. Treatment with resveratrol also decreased expression of ROS and tyrosine hydroxylase (TH). Conclusion: Resveratrol within the PVN attenuates hypertension via the SIRT1/NF-κB pathway to decrease ROS and restore the balance of excitatory and inhibitory neurotransmitters.

## 1. Introduction

Hypertension is a serious threat to human health due to its high morbidity and mortality rate [1]. The hypothalamic paraventricular nucleus (PVN) influences cardiovascular activity by secreting various neural and humoral factors [2,3]. The PVN is crucial in controlling sympathetic nerve activity (SNA) and contributes to blood pressure regulation [4]. Resveratrol is a kind of polyphenol compound, which mostly exists in grapes and red wine [5,6]. Resveratrol exhibits obvious antioxidant and anti-inflammatory effects in the treatment of hypertension [7,8,9]. Recent studies have also confirmed that resveratrol is an effective activator of silent mating-type information regulation 2 homolog-1 (sirtuin1, SIRT1) [10,11]. SIRT1, a deacetylase, functions as a silent information regulator in mammals [12]. It is a highly conserved protein with myocardial protective effects, such as improved endothelial function, enhanced cardiac function, and reduced atherosclerosis [13,14]. However, it remains unclear whether SIRT1 has any effect on hypertension in the central nervous system.

An increase in oxidative stress is not only a signature pathological event in cardiovascular disease, but also an important factor affecting SNA in hypertension [15]. One of the main characteristics of oxidative stress is massive production of reactive oxygen species (ROS) [16]. The redox pathway related to the NAD(P)H oxidase (NOX) family is a critical mechanism leading to increased ROS levels in chronic cardiovascular diseases [17]. NOX2 and NOX4 are the two main components of the NOX family [18]. The authors of [19] found that ROS production in the PVN and sympathetic activity increased in Aldo/NaCl induced hypertensive mice, whereas injection of AdsiRNA-NOX2 or AdsiRNA-NOX4 into the PVN significantly lowered blood pressure. Zhu found that microinjection of ROS scavengers or NOX inhibitors into the PVN attenuated SNA and thereby lowered blood pressure [20]. Similarly, our laboratory found that administration of tempol via the PVN significantly repressed renal sympathetic nerve activity (RSNA), and lowered plasma norepinephrine (NE) and arterial pressure [21].

Nuclear factor κB (NF-κB) is an extensively studied nuclear transcription factor [22], which includes five subunits: C-Rel, NF-κB1 (p50/p105), NF-κB2 (p52/p100), RelA (p65), and RelB [23]. The activity of NF-κB in the PVN is closely related to the enhancement of SNA. Inhibiting NF-κB activities greatly reduced ROS and SNA in hypertension [24] and myocardial infarction rat models [25,26]. The authors of [27] found that NF-κB activity increased in myeloid-specific SIRT1 knockout mice, suggesting that SIRT1 in bone marrow may inhibit NF-κB activation. In addition, the authors of [28] found that SIRT1 can interfere with cell apoptosis and improve cell survival by deacetylate NF-κB, and thus play a neuroprotective role in the development of neurodegenerative diseases. For the purposes of this study, we hypothesized that, in cases of hypertension, SIRT1 would regulate ROS levels through the NF-κB pathway in the PVN.

Tyrosine hydroxylase (TH) and glutamate decarboxylase 67 (GAD67) are the rate-limiting enzymes for the production of excitatory and inhibitory neurotransmitters in the central nervous system [29,30]. The production of excitatory neurotransmitters, such as dopamine and NE in the brain, depends on TH [31]. GAD67 promotes the degradation of glutamate (Glu) and accelerates the production of inhibitory neurotransmitter γ-aminobutyric acid (GABA) [30]. Our laboratory studies and those of others have shown increased TH expression and decreased GAD67 expression in the PVN during hypertension [32] and heart failure [33], causing an imbalance between excitatory and inhibitory neurotransmitters [34]. In addition, suppressing NF-κB activities in the PVN reduces RSNA during heart failure by restoring the balance of excitatory and inhibitory neurotransmitters [35].

In summary, this study explored the hypothesis that administration of resveratrol in the PVN would attenuate high blood pressure via the SIRT1/NF-κB pathway, by regulating ROS and neurotransmitters during hypertension.

## 2. Materials and Methods

### 2.1. Animals

Healthy male Sprague-Dawley rats weighing 275–300 g were provided from the animal center of Xi’an Jiaotong University. The Animal Care and Use Committee of the same institution approved the animal protocols (No. 2020-63). All rats were kept in a room with constant temperature and humidity, and a 12-hour light-dark cycle, and allowed access to normal rat chow and tap water ad libitum. Procedures involved in this study were performed in accordance with the Guide for Care and Use of Laboratory Animals published by the US National Institutes of Health (NIH publication, 8th edition, 2011).

### 2.2. PVN Cannula Implantation

All rats were implanted bilaterally with PVN with sterilizing cannulas after anesthetizing with isoflurane in line with methods previously cited in the literature [21].

### 2.3. Preparation and Grouping of Animal Models

After the bilateral PVN cannulas were implanted, all animals were allowed to convalesce for seven days. All rats were then shaved at the surgical site, gas anesthetized with isoflurane, and placed in the left decubitus position. The abdominal surgery was performed to isolate the right renal artery. A silk thread (2-0 specification) was passed through the right renal artery, and a 0.25 mm silver acupuncture needle was placed above the isolated artery, tightening the artery with the needle and causing immediate ischemia of the right kidney. The ischemic state gradually recovered after we carefully withdrew the acupuncture needles. Suturing layer by layer with surgical sutures resulted in stenosis of the right renal artery, constructing a two-kidney one-clip (2K1C) hypertensive animal model. The rats in the sham surgery (SHAM) group were threaded but not ligated, and the surgical mouth was directly sutured. The rats were assigned to four groups at random: (i) SHAM + PVN vehicle; (ii) SHAM + PVN resveratrol; (iii) 2K1C + PVN vehicle; and (iv) 2K1C + PVN resveratrol. Vehicle (artificial cerebrospinal fluid, aCSF, 0.4 μL) or resveratrol (a SIRT1 agonist, 160 μmol/L, 0.4 μL) [36] was then injected into the bilateral PVN of rats each day for four consecutive weeks. Penicillin was administered for three days in doses according to weight, and the animals were observed daily.

### 2.4. Measurement of Blood Pressure

In line with previously cited methods [37], the non-anesthetized rats were placed on a thermostatically controlled heating plate and heated up to an ambient temperature of 36 ℃ for 15 min, and systolic blood pressure (SBP) was recorded by a non-invasive tail-cuff instrument (BP-300, Chengdu Techman Software Co., Ltd., Chengdu, China) every four days. A seven-day pre-training session before the experiment was necessary for all rats to fully adapt to the measurement procedure.

### 2.5. Collection of Blood and Tissue Samples

Animals were anesthetized with isoflurane at the end of 28th day. Plasma specimens and brain tissue were collected and stored at −80 °C for future analysis [38].

### 2.6. Immunofluorescence Staining

According to the specific location of the PVN in the rat brain atlas, transverse sections with a thickness of 18 μm were obtained and immunofluorescence staining was performed [34]. The primary antibodies were SIRT1 (#7475S, CST, MA, USA, 1:400 dilution), NOX4 (ab-133303, Abcam, Cambridge, UK, 1:1000 dilution), TH (sc-25269, Santa Cruz, TX, USA, 1:50 dilution), and GAD67 (ab-26116, Abcam, Cambridge, UK, 1:200 dilution). Dihydroethidium (DHE, Molecular Probes, Eugene, OR, USA) was used to examine ROS generation.

### 2.7. Western Blotting

In line with previously cited methods [32], the PVN of rats was fragmented by ultrasound and corresponding tissue proteins were extracted for Western blotting detection. The primary antibodies for SIRT1 (#7475S, CST, MA, USA, 1:1000 dilution), copper-zinc superoxide dismutase (SOD1, WL01846, Wanleibio, Shenyang, China, 1:1000 dilution), and β-actin (sc-8432, Santa Cruz, TX, USA, 1:2000 dilution) were bought. Using the wet transfer method, the related proteins were transferred to a PVDF membrane. The strips were then sealed with skimmed milk (4%) and incubated with primary antibodies at four degrees overnight. The following day, they were combined with the corresponding secondary antibody (GB23301, GB23303, Servicebio, Wuhan, China, 1:5000 dilution), then added with chemiluminescence reagent, and protein content was detected.

### 2.8. Enzyme-Linked Immunosorbent Assay (ELISA)

Levels of plasma NE (H096, Nanjing Jiancheng, Nanjing, China), NOX (ab186031, Abcam, Cambridge, UK), and NF-κB p65 (R0674c, elabscience, Wuhan, China) in rat PVN were quantitatively detected using an ELISA kit at 460 nm [32]. Values were then calculated using a spectrophotometer.

### 2.9. Statistical Analysis

Our data were described in terms of mean ± SEM and two-way ANOVA were performed followed by a post-hoc Tukey test. Blood pressure data were analyzed with repeated measures ANOVA. *p* values lower than 0.05 were regarded as statistically significant.

## 3. Result

### 3.1. Blood Pressure

**Figure 1:**
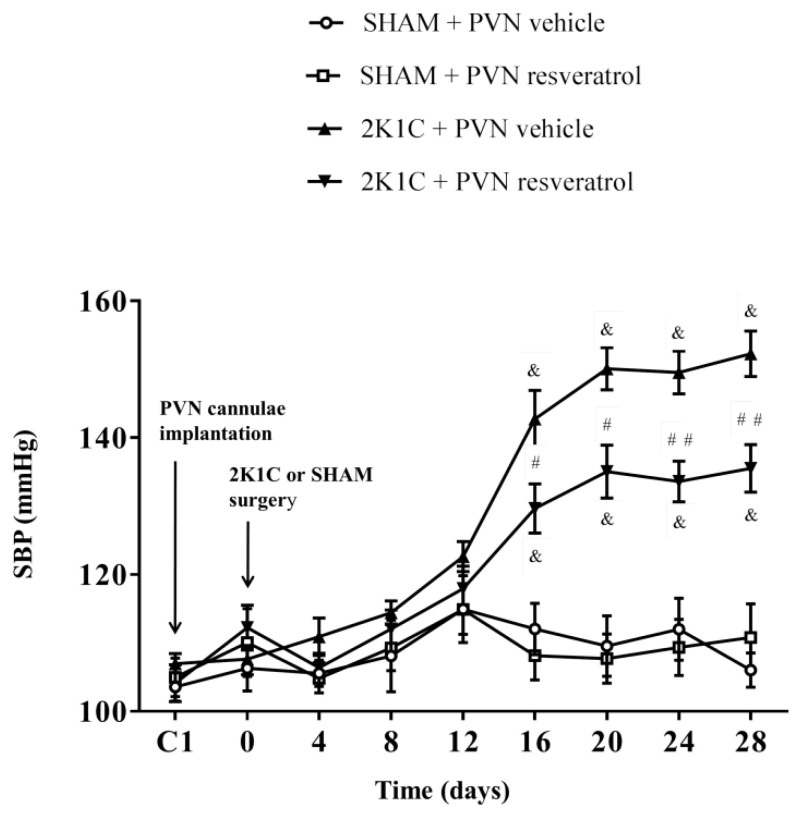
The effects of resveratrol on SBP. Values are the mean ± SEM. ^&^ *p* < 0.001 vs. SHAM groups; ^#^ *p* < 0.05 or ^##^ *p* < 0.01, relatively to 2K1C + PVN vehicle, *n* = 5.

As shown in Figure 1, in contrast to SHAM rats, SBP in 2K1C rats significantly increased from the 16th day and remained increased (^&^ *p* < 0.001). However, compared with 2K1C + PVN vehicle rats, SBP was significantly reduced from the 16th day to the end in 2K1C + PVN resveratrol rats (Figure 1, ^#^ *p* < 0.05, ^##^ *p* < 0.01). The basal SBP in each group was similar.

### 3.2. Plasma NE

**Figure 2:**
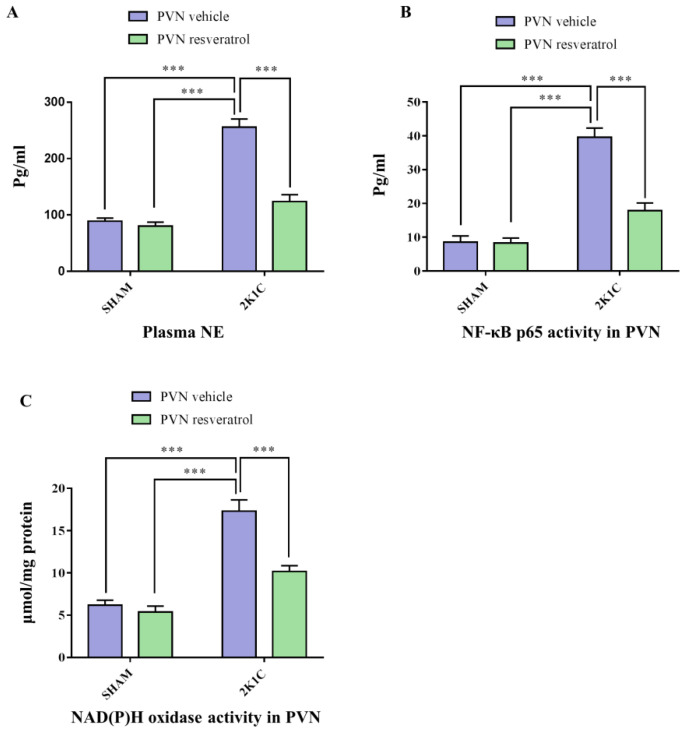
The effects of resveratrol on the plasma levels of NE, and PVN levels of NOX and NF-κB activation. (**A**) Statistical analysis of NE; (**B**) Statistical analysis of NF-κB p65 activity; and (**C**) Statistical analysis of NOX. Values are the mean ± SEM. *** *p* < 0.001, *n* = 4–5.

As shown in Figure 2A, in contrast with SHAM animals, hypertensive rats presented a higher level of plasma NE (*p* < 0.001). After four weeks of infusion of resveratrol, compared with 2K1C + PVN vehicle rats, 2K1C + PVN resveratrol rats had an attenuated level of plasma NE (Figure 2A, *p* < 0.001).

### 3.3. SIRT1 Expression in PVN

**Figure 3:**
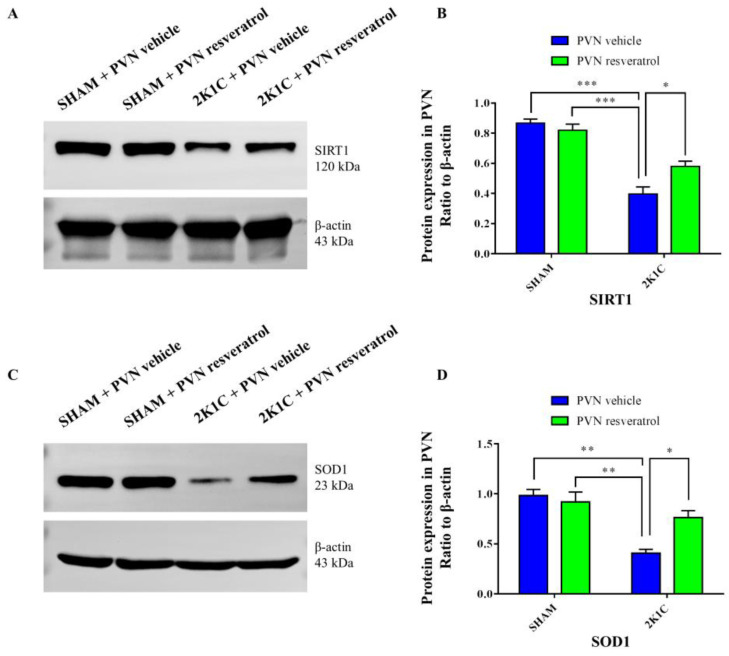
The effects of resveratrol on SIRT1 and SOD1 protein expression in PVN. (**A**) Representative immunoblots of SIRT1 and β-actin; (**B**) densitometry protein expression of SIRT1; (**C**) immunoreactive bands of SOD1 and β-actin; and (**D**) statistic of SOD1 protein expression. Values are the mean ± SEM. * *p* < 0.01, ** *p* < 0.01, *** *p* < 0.001, *n* = 3.

**Figure 4:**
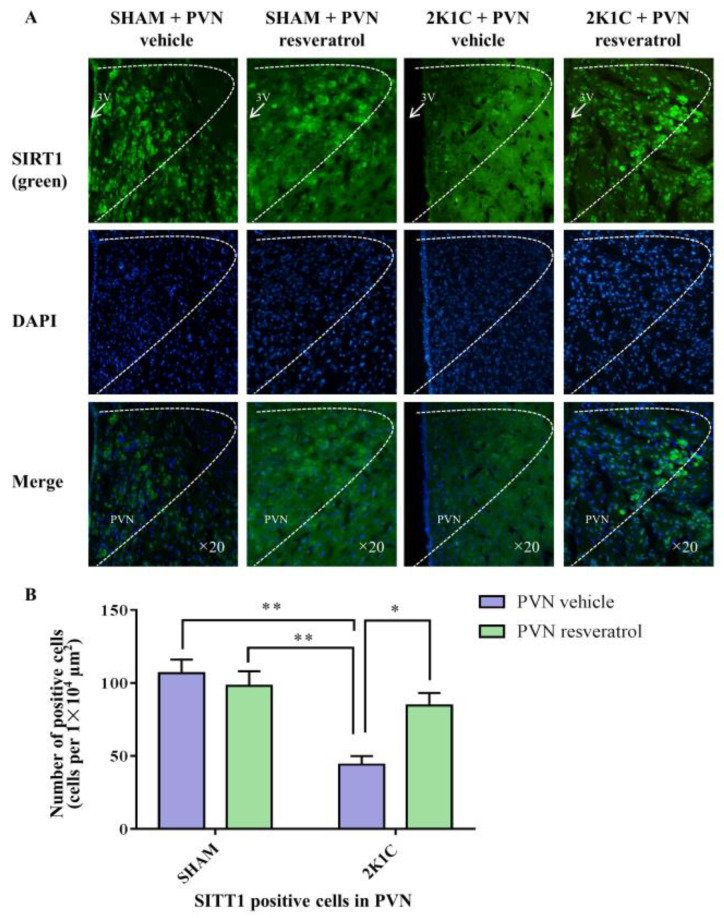
The effects of resveratrol on SIRT1 expression in PVN. (**A**) Immunofluorescence staining of SIRT1; (**B**) statistics of SIRT1 positive cells. 3V: third ventricle. Values are the mean ± SEM. * *p* < 0.05, ** *p* < 0.01, *n* = 4.

As shown in Figure 3A,B and Figure S1, in contrast with the SHAM rats, SIRT1 protein expression in 2K1C rats were reduced significantly (*p* < 0.001). After four weeks of PVN infusion of resveratrol, SIRT1 protein expression was increased (Figure 3A,B and Figure S1, *p* < 0.05). As shown in Figure 4A,B, in contrast with the SHAM rats, the number of SIRT1 positive cells of hypertensive rats was significantly reduced (Figure 4A,B, *p* < 0.01). Four weeks of PVN infusion of resveratrol effectively increased the number of SIRT1 positive cells (*p* < 0.05).

### 3.4. NF-κB Activity in the PVN

As shown in Figure 2B, in contrast with the SHAM rats, the NF-κB p65 activity in 2K1C rats was significantly increased (*p* < 0.001). When rats were infused with resveratrol for 28 days, NF-κB p65 activity was decreased (Figure 2B, *p* < 0.001).

### 3.5. NAD(P)H Oxidase Activity in the PVN

As shown in Figure 2C, in contrast with the SHAM rats, the NOX activity in hypertensive rats was significantly increased (*p* < 0.001). After four weeks of infusion with resveratrol, NOX activity was decreased (Figure 2C, *p* < 0.001).

### 3.6. ROS Production in PVN

**Figure 5:**
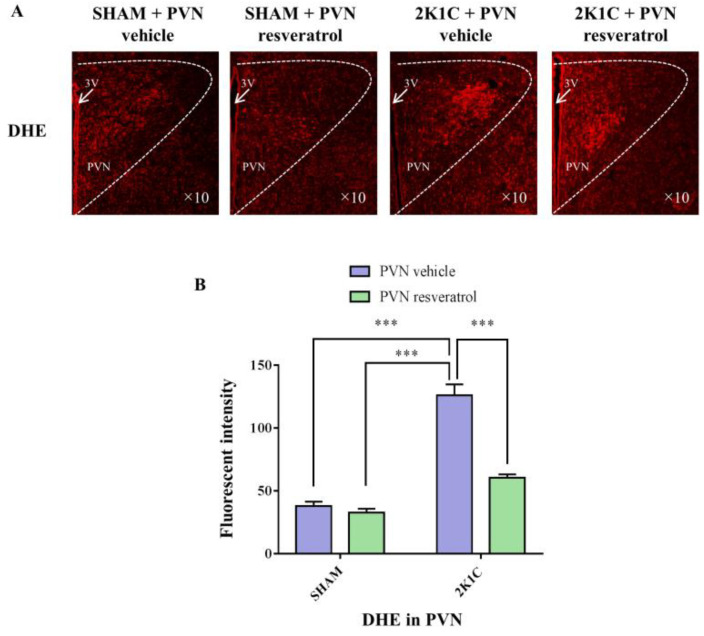
The effects of resveratrol on ROS in PVN. (**A**) Immunofluorescence staining of reactive oxygen species (red fluorescence, ×10) and (**B**) densitometric analysis of DHE staining. 3V: third ventricle. Values are the mean ± SEM. *** *p* < 0.001, *n* = 4.

As shown in Figure 5, ROS levels in the hypertensive rats were higher than in the SHAM rats (*p* < 0.001). When rats were infused with the SIRT1 agonist, resveratrol, for 28 days, the level of ROS was reduced (Figure 5, *p* < 0.001).

### 3.7. SOD1 Protein Expression in the PVN

As shown in Figure 3C,D and Figure S2, in contrast with the SHAM rats, SOD1 protein expression in 2K1C rats significantly decreased (*p* < 0.01). When rats were infused with resveratrol for 28 days, SOD1 expression was increased (Figure 3C,D and Figure S2, *p* < 0.05).

### 3.8. NOX4 Expression in PVN

**Figure 6:**
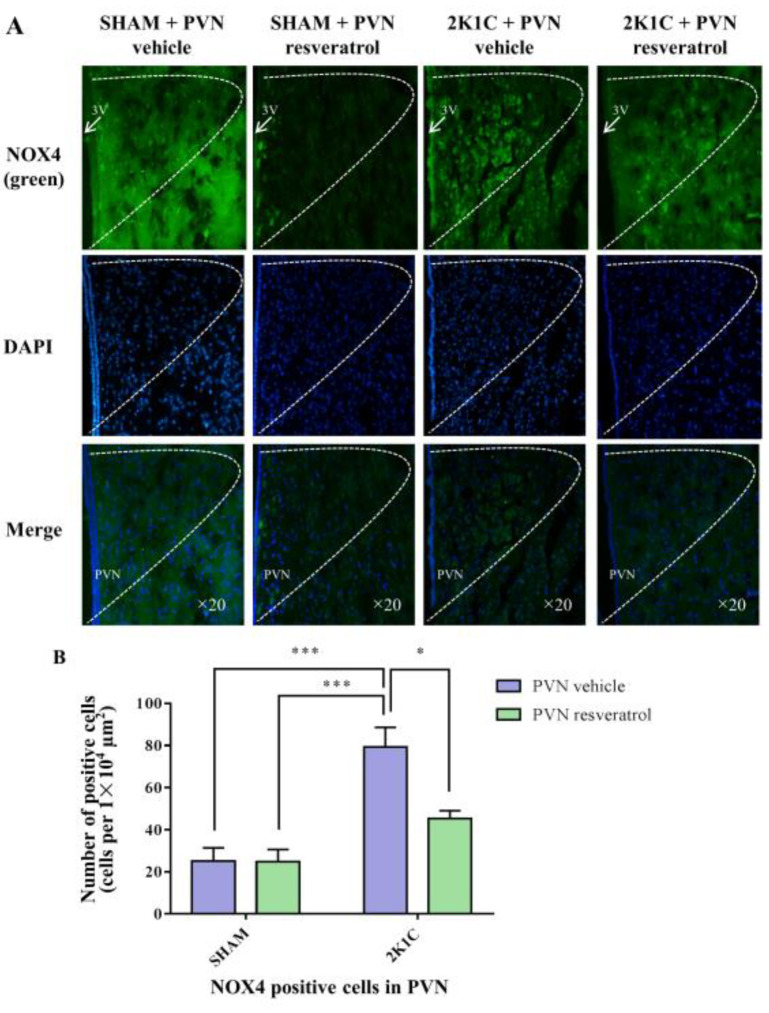
The effects of PVN resveratrol on NOX4 expression. (**A**) Immunofluorescence staining of NOX4 (green fluorescence, ×20) and (**B**) statistics of NOX4 positive cells. 3V: third ventricle. Values are the mean ± SEM. * *p* < 0.05, *** *p* < 0.001, *n* = 4.

As shown in Figure 6, in contrast with the SHAM rats, the number of NOX4 positive cells in 2K1C rats significantly increased (*p* < 0.001). When rats were infused with resveratrol for four weeks, the number of NOX4 positive cells was reduced (Figure 6, *p* = 0.0161).

### 3.9. TH Expression in the PVN

**Figure 7:**
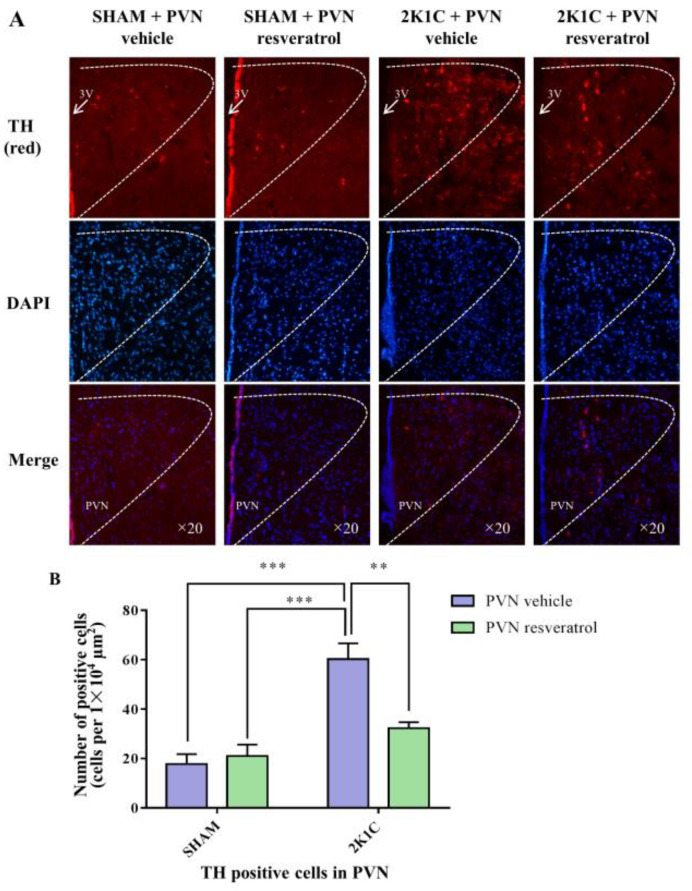
The effects of PVN resveratrol on TH expression. (**A**) Immunofluorescence staining of TH (red fluorescence, ×20) and (**B**) statistics of TH positive cells. 3V: third ventricle. Values are the mean ± SEM. ** *p* < 0.01, *** *p* < 0.001, *n* = 4.

As shown in Figure 7, in contrast with the SHAM rats, the number of TH positive cells in 2K1C rats significantly increased (*p* < 0.001). When rats were infused with resveratrol for 28 days, the number of TH positive-cells was reduced (Figure 7, *p* = 0.0048).

### 3.10. GAD67 Expression in the PVN

**Figure 8:**
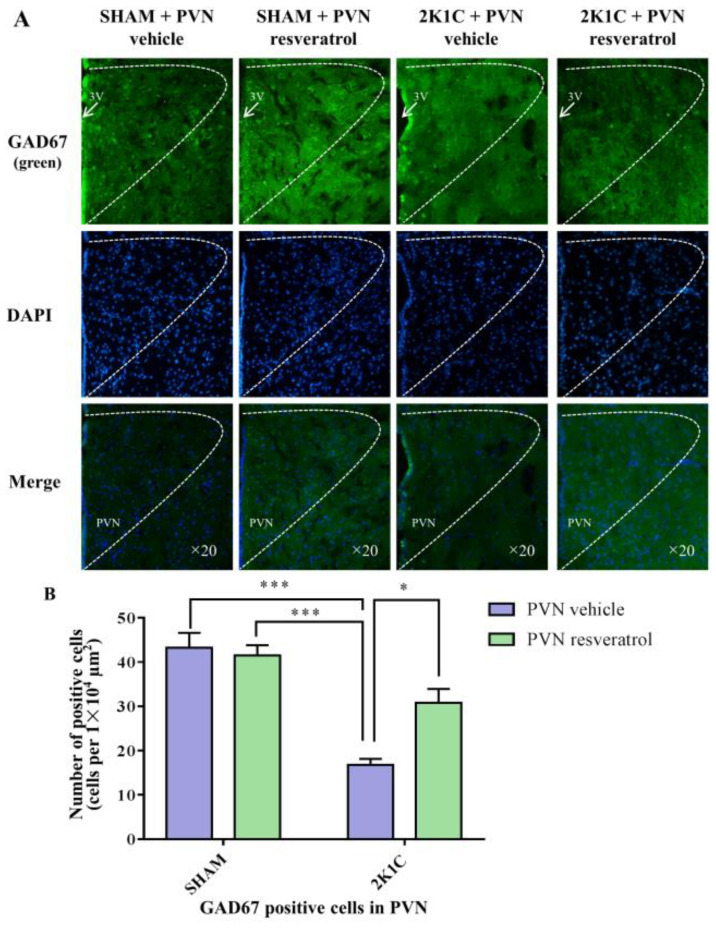
The effects of PVN resveratrol on GAD67 expression. (**A**) Immunofluorescence staining of GAD67 (green fluorescence, ×20) and (**B**) statistics of GAD67 positive cells. 3V: third ventricle. Values are the mean ± SEM. * *p* < 0.05, *** *p* < 0.001, *n* = 4.

In comparison with the SHAM rats, the number of GAD67 positive cells in 2K1C rats significantly decreased (Figure 8, *p* < 0.001). When rats were infused with resveratrol for 28 days, the number of GAD67 positive cells was increased (Figure 8, *p* = 0.0142).

## 4. Discussion

A number of recent studies have reported that ROS in the PVN is crucial to the pathogenesis of cardiovascular diseases [25,39]. High ROS levels in the PVN lead to overstimulation of peripheral SNA [40]. Activation of the NOX family, particularly NOX2 and NOX4, is closely associated with ROS production in the PVN [41]. SOD1 is an important antioxidant enzyme which inhibits ROS production [32]. Some studies have shown that elevated NOX activity and ROS production in the PVN stimulates abnormal peripheral SNA in high-salt or Ang II-induced hypertension rats [38,41]. Blocking the excessive production of ROS in PVN can inhibit SNA in hypertensive rats [40]. Zhou found that inhibiting SIRT1 increased ROS production in the aortic endothelial cells and aortic vascular smooth muscle cells of mice [42].

The authors of [43] found evidence that resveratrol, an anti-inflammatory and antioxidant substance, is an effective activator of SIRT1 [10]. In addition, SIRT1 overexpression in the rostral ventrolateral medulla (RVLM), another central region that regulates sympathetic activity, led to lower blood pressure and reduced sympathetic outflow by lowering ROS levels in spontaneously hypertensive rats [44,45]. In accordance with these findings, our study found that PVN infusion of resveratrol increased SIRT1 and SOD1 protein expression and decreased the expression of NOX4 and ROS in 2K1C hypertensive rats, suggesting that activated SIRT1 exerts anti-oxidative stress effects in other central regions besides the RVLM. However, a study by Liu et al. showed that SIRT1 expression was increased in the arcuate nucleus of the hypothalamus (ARC) in obese hypertensive rats, and knockdown of SIRT1 in the ARC could decrease the RSNA and blood pressure in leptin-induced obese hypertension [46]. The above results suggest conflicting roles of SIRT1 in PVN and ARC in different hypertensive models. Further investigation is needed to fully understand the effects of SIRT1 mechanisms upon blood pressure regulation in different nuclei of the brain.

Researchers have also found that NF-κB activities in the PVN are closely associated with the intensity of SNA in hypertensive rats [24,47]. NF-κB and oxidative stress in PVN play crucial roles in the pathogenesis of hypertension [38,48]. SIRT1 can directly act on NF-κB and reduce the acetylation level of p65 subunit Lys^310^, thereby inhibiting the transcription activity of NF-κB [49]. Knockout of SIRT1 can lead to excessive NF-κB activity [50]. SIRT1 can also suppress NF-κB-mediated oxidative stress, including NOX, whereas loss of SIRT1 can lead to the hyperacetylation of NF-κB and the enhancement of oxidative stress [51]. Our study revealed that PVN injection with resveratrol lowered levels of NF-κB p65 activity, and reduced NOX4 and ROS expression in 2K1C hypertensive rats.

NE, Glu, and GABA are important neurotransmitters in the PVN that regulate SNA [52]. GABA has been shown to elicit sympatho-inhibitory responses in the PVN [32]. NE is a neurotransmitter secreted from the terminals of noradrenergic nerve fibers. Its content in peripheral blood can reflect the excited state of sympathetic nerves, which is increased in both peripheral and central systems in heart failure rat models [53]. Glu is a major excitatory neurotransmitter, and l-glutamate microinjected into the PVN can cause increased blood pressure [54]. Megan et al. found that injection of the GABA-A receptor agonist muscarine into the PVN significantly reduced RSNA and mean arterial pressure of Ang II- and salt-induced hypertensive rats [55]. An accumulating body of evidence suggests that the loss of balance in neurotransmitter levels in the PVN contributes to abnormal sympathetic activity in heart failure [33,53] and hypertension [34]. Previous studies of our group showed that NF-κB activation and TH expression increased in the PVN of hypertension [32,39] or heart failure rat models, whereas inhibition of NF-κB decreased TH expression in the PVN of heart failure rats [35]. In this study, PVN injection with resveratrol reduced TH levels but increased GAD67 levels. These data suggest that SIRT1 activation in the PVN restores balance in neurotransmitter expressions by acting on NF-κB, thereby affecting SNA and blood pressure.

## 5. Conclusions

Our results showed that administration of resveratrol in the PVN decreased ROS expression, restored neurotransmitter balance, and subsequently attenuated high blood pressure in 2K1C rats via the SIRT1/NF-κB pathway.

## Data Availability

All relevant data are within the manuscript and its Supporting Information files.

## Supplementary Materials

The following supporting information can be downloaded at: https://www.mdpi.com/article/10.3390/nu14194177/s1, Figure S1: Resveratrol increased SIRT1 protein expression of 2K1C rats; Figure S2: Resveratrol increased SOD1 protein expression of 2K1C rats.

## Abbreviations

PVN	Hypothalamic Paraventricular Nucleus
SNA	sympathetic nerve activity
SIRT1	Silent Mating type information regulation 2 homolog-1
ROS	reactive oxygen species
NOX	NAD(P)H oxidase
RSNA	renal sympathetic nerve activity
NF-κB	nuclear factor-kappa B
TH	Tyrosine hydroxylase
GAD67	Glutamate decarboxylase 67
NE	norepinephrine
Glu	glutamate
GABA	γ-aminobutyric acid
2K1C	two-kidney one-clamp
SHAM	sham operation
aCSF	artificial cerebrospinal fluid
SBP	systolic blood pressure
DHE	Dihydroethidium
RVLM	rostral ventrolateral medulla
ARC	arcuate nucleus of the hypothalamus
