# Supplementary material for: Resveratrol in the Hypothalamic Paraventricular Nucleus Attenuates Hypertension by Regulation of ROS and Neurotransmitters
Source: Nutrients. 2022 Oct 7;14(19):4177. doi: 10.3390/nu14194177 (PMC9573276; doi:10.3390/nu14194177)
Supplement: Supplementary file 1 [file nutrients-14-04177-s001.zip › nutrients-1874751-supplementary.pdf]

Western blotting Gels:

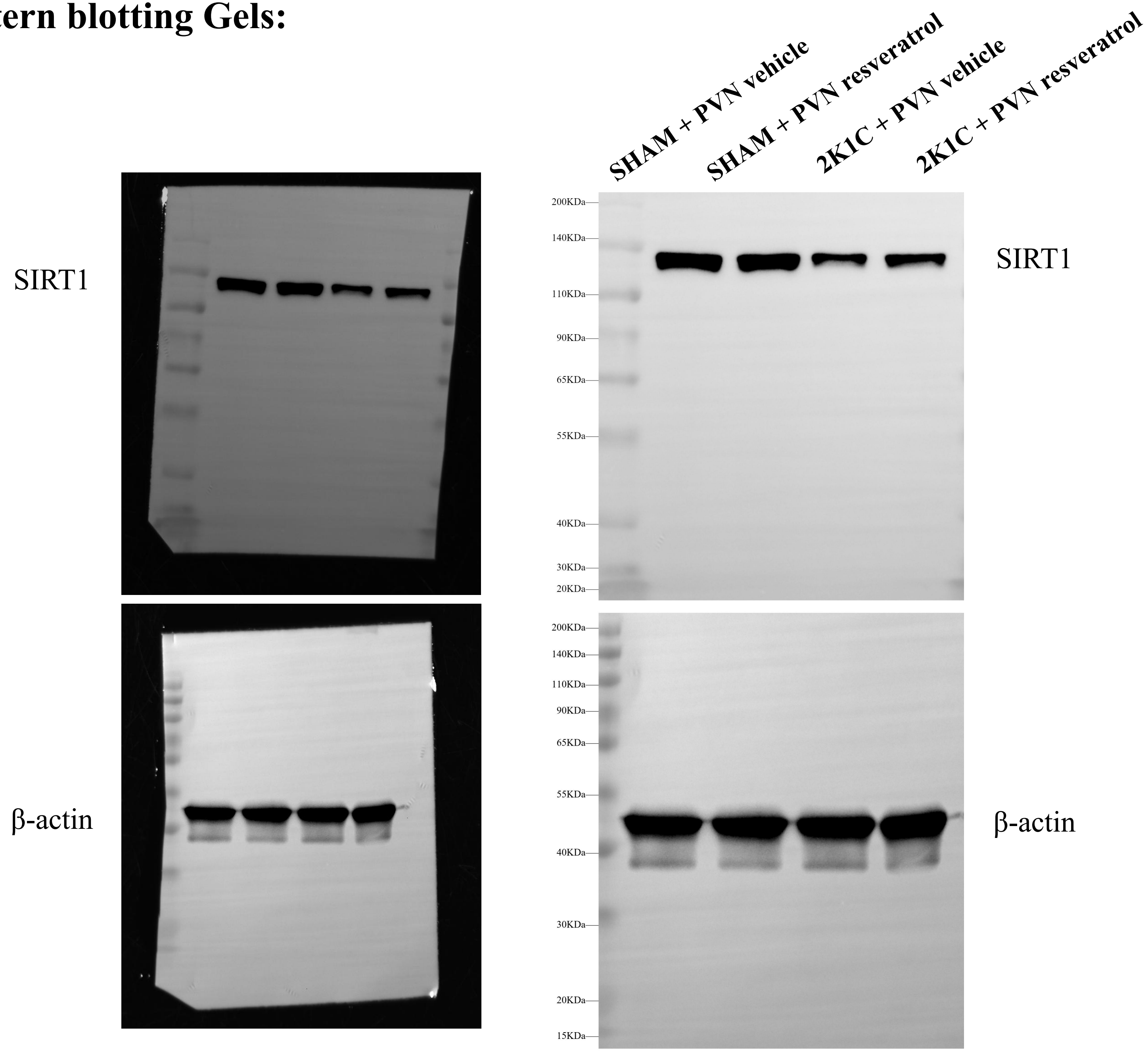

Figure S1: Resveratrol increased SIRT1 protein expression in the PVN of 2K1C rats.

Western blotting Gels:

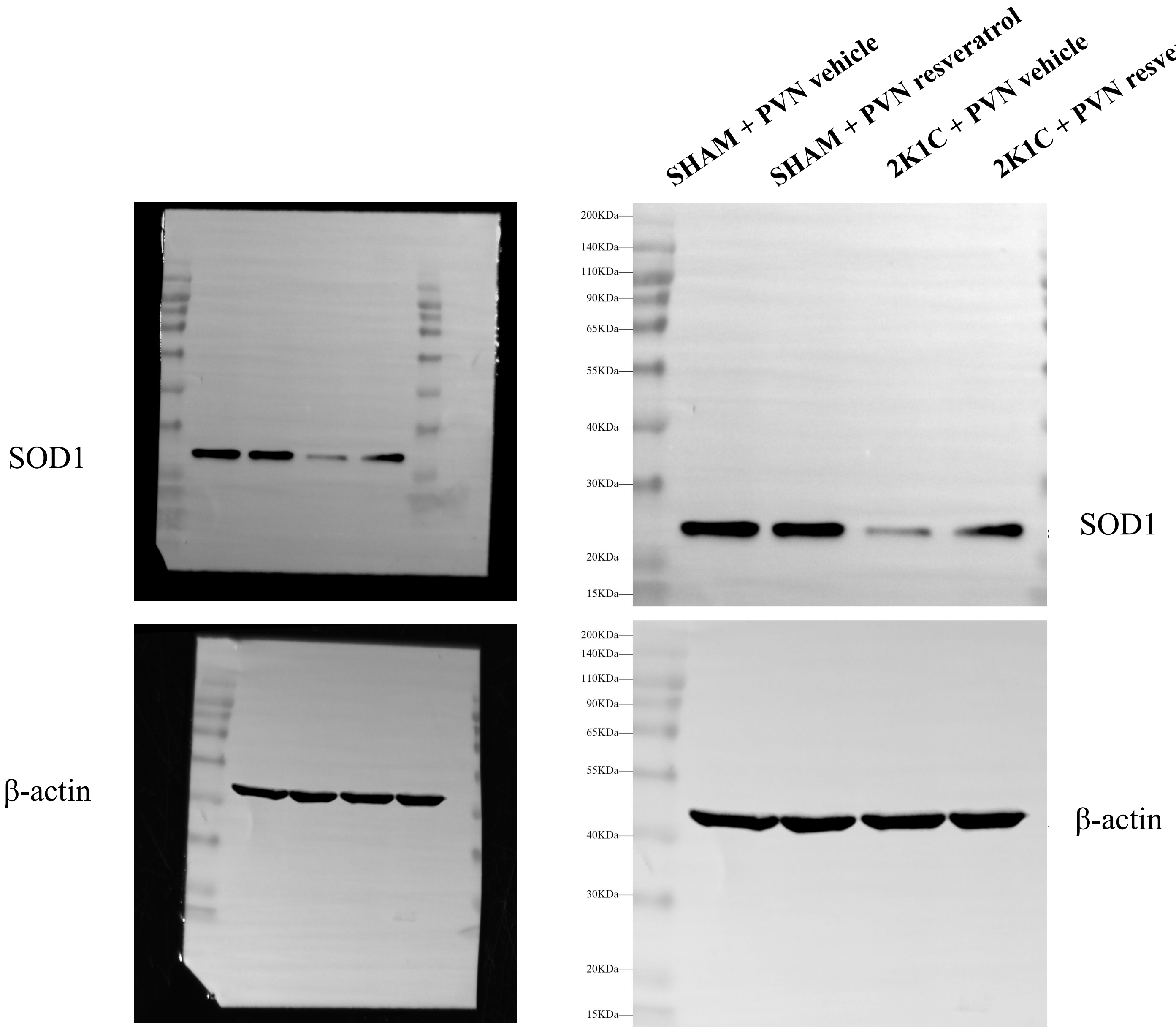

Figure S2: Resveratrol increased SOD1 protein expression in the PVN of 2K1C rats.
